# Integrative transcriptomic and network pharmacology analysis reveals the neuroprotective role of BYHWD through enhancing autophagy by inhibiting Ctsb in intracerebral hemorrhage mice

**DOI:** 10.1186/s13020-023-00852-3

**Published:** 2023-11-13

**Authors:** Yiqing Cai, Zhe Yu, Xueping Yang, Weikang Luo, En Hu, Teng Li, Wenxin Zhu, Yang Wang, Tao Tang, Jiekun Luo

**Affiliations:** 1 Department of Integrated Traditional Chinese and Western Medicine, Institute of Integrative Medicine, Xiangya Hospital, Central South University, Changsha, 410008 Hunan People’s Republic of China; 2 National Clinical Research Center for Geriatric Disorders, Xiangya Hospital, Central South University, Changsha, 410008 Hunan People’s Republic of China; 3 National Regional Center for Neurological Diseases, Xiangya Hospital, Central South University Jiangxi, Nanchang, 330000 Jiangxi People’s Republic of China

**Keywords:** Intracerebral hemorrhage, Buyang Huanwu Decoction, Transcriptomic, Network pharmacology, Autophagy

## Abstract

**Background:**

In this study, we aimed to combine transcriptomic and network pharmacology to explore the crucial mRNAs and specific regulatory molecules of Buyang Huanwu Decoction (BYHWD) in intracerebral hemorrhage (ICH) treatment.

**Methods:**

C57BL/6 mice were randomly divided into three groups: sham, ICH, and BYHWD. BYHWD (43.29 g/kg) was administered once a day for 7 days. An equal volume of double-distilled water was used as a control. Behavioural and histopathological experiments were conducted to confirm the neuroprotective effects of BYHWD. Brain tissues were collected for transcriptomic detection. Bioinformatics analysis were performed to illustrate the target gene functions. Network pharmacology was used to predict potential targets for BYHWD. Next, transcriptomic assays were combined with network pharmacology to identify the potential differentially expressed mRNAs. Immunofluorescence staining, real-time polymerase chain reaction, western blotting, and transmission electron microscopy were performed to elucidate the underlying mechanisms.

**Results:**

BYHWD intervention in ICH reduced neurological deficits. Network pharmacology analysis identified 203 potential therapeutic targets for ICH, whereas transcriptomic assay revealed 109 differentially expressed mRNAs post-ICH. Among these, cathepsin B, ATP binding cassette subfamily B member 1, toll-like receptor 4, chemokine (C–C motif) ligand 12, and baculoviral IAP repeat-containing 5 were identified as potential target mRNAs through the integration of transcriptomics and network pharmacology approaches. Bioinformatics analysis suggested that the beneficial effects of BYHWD in ICH may be associated with apoptosis, animal autophagy signal pathways, and PI3K-Akt and mTOR biological processes. Furthermore, BYHWD intervention decreased Ctsb expression levels and increased autophagy levels in ICH.

**Conclusions:**

Animal experiments in combination with bioinformatics analysis confirmed that BYHWD plays a neuroprotective role in ICH by regulating Ctsb to enhance autophagy.

## Background

Spontaneous nontraumatic intracerebral hemorrhage(ICH) is a serious subtype that accounts for approximately 10–15% of all types of strokes [1]. The overall incidence of ICH is 24.6 per 100 000 person-years, with a mortality rate of 40% [2, 3]. Moreover, ICH presents acutely and progresses rapidly, resulting in hemiplegia and altered consciousness, thus imposing a substantial global clinical and economic burden [4, 5]. Current treatments for ICH involve surgical and conservative medical approaches [6]. However, the results of multiple clinical trials have indicated that surgical interventions did not significantly improve the adverse outcomes in ICH [7]. Furthermore, no specific therapeutic medication has been approved for ICH treatment, and supportive therapy remains the primary approach for patients. Despite increased attention to ICH management, the available treatment options and effective targets are currently limited [8].

Brain injuries resulting from ICH encompass intricate pathophysiological processes, categorised as either primary or secondary brain injury based on pathological patterns [9]. Primary injury refers to the local tissue damage caused by an intracerebral haematoma, primarily occurring within minutes to hours after the onset of the disease [9]. Secondary brain injury is primarily induced by inflammation, oxidative stress, and excitotoxicity, all of which can initiate substantial neuronal cell death [10]. In such instances, the extensive cellular and tissue debris cannot be timely cleared, leading to the accumulation of detrimental substances that can result in persistent neurological dysfunction [11–17]. The neurological deficit caused by brain injury may induce autophagy. Autophagy plays a critical role in the elimination of cellular debris and waste following injury, thereby facilitating neuronal repair in nervous system diseases [18–23].

Buyang Huanwu Decoction (BYHWD) is widely recognised as a clinically effective formula for treating various types of strokes. Previous systematic reviews and meta-analyses have consistently demonstrated the efficacy and safety of BYHWD for treating ICH. [24, 25]. As a neuroprotective agent, BYHWD modulates the non-classical NF-κB pathway, attenuating the inflammatory response following ICH [23]. Furthermore, previous experimental studies have indicated that BYHWD promotes lactate accumulation and activates the HIF-1α/VEGF signal pathway to induce angiogenesis post-ICH [24]. Additionally, BYHWD downregulates the expression of leukemia inhibitory factors, thereby reducing glial scar formation induced by ICH [26–29]. Nonetheless, the potential targeting of autophagy by BYHWD warrants further investigation.

The mechanisms underlying the effects of BYHWD in ICH remain unclear owing to the complexity of herbal compounding. Transcriptomic technology offers a valuable tool for gaining a fundamental understanding of molecular regulation and exploring novel drug targets [30]. Transcriptomic technology has been employed to validate changes in the expression of specific RNAs following pharmacological interventions in brain hemorrhage [31, 32]. Network pharmacology technology is an intuitive and comprehensive approach for identifying core targets [33]. In this study, we combined transcriptomics and network pharmacology analyses to explore crucial target mRNAs and specific regulatory molecules associated with BYHWD in ICH treatment. This research is expected to provide deeper insights into the transcriptional-level effects of BYHWD in ICH treatment.

## Methods

### BYHWD preparation

The original prescription of BYHWD is available in the ‘TCM Prescriptions Dictionary’, which lists the following seven Chinese herbs: *Astragalus mongholicus* Bunge, *Angelicae sinensis* (Oliv.) Diels, *Ligusticumi* chuanxiong Hort, *Paeonia lactiflora* Pall, *Prunus persica (L.)* Batsch, *Carthamus tinctorius L*, and *Pheretima aspergillum* (E. Perrier) (Table 1). The raw herbs were sourced from the Xiangya Hospital of Central South University (Changsha, China). The authenticity of the plant materials was confirmed by Professor Suiyu Hu from the Department of Chinese Herbal Medicine at Central South University in Changsha, China. Voucher specimens of the plants were deposited at Xiangya Hospital, affiliated with Central South University (Changsha, China). These herbs were decocted twice and dissolved in distilled water to a final concentration. Based on the preliminary experimental results, mice with ICH were intragastrically administered 43.29 g/kg raw herbs in the BYHWD group.

**Table 1:** Composition and corresponding ratio of BYHWD

Herb	Chinese name	Medicinal part	Dosage
*Astragalus mongholicus* Bunge	Huang Qi	Root	60
*Angelicae sinensis* (Oliv.), Diels	Dang Gui	Root	9
*Ligusticumi chuanxiong* Hort	Chuan Xiong	Rhizome	6
*Paeonia lactiflora* Pall	Chi Shao	Root	9
*Prunus persica (L.)* Batsch	Tao Ren	Seed	9
*Carthamus tinctorius L*	Hong Hua	Flower	9
*Pheretima aspergillum* (E. Perrier)	Di Long	Whole animal	9

#### Animals

Adult male C57BL/6 specific pathogen-free mice (25–30 g) were provided by the Animal Experimental Center of Central South University, China. The animals were housed under controlled conditions (temperature, 23 °C ± 2 °C; relative humidity, 50% ± 10%; 12 h light/darkness) and had free access to standard nutrition throughout the study period.

#### Experimental groups

The mice were randomly divided into the sham, ICH, and BYHWD groups. Before surgery, the animals were deeply anesthetized by sodium pentobarbital solution (0.3%, 0.22 mL/10 g). Then the tails of the mice were transected at a 3-mm diameter, and 15 μL blood was collected. Subsequently, the mice were mounted on a stereotactic headframe in the prone position. A scalp incision was made, and a small cranial burr was drilled in the skull near the right coronal suture. Blood was injected slowly (over 5 min) via a sterile microliter syringe into the right globus pallidus, according to the following coordinates relative to bregma: 0.5 mm posterior, 2 mm lateral, and 4 mm ventral to the cortical surface. As a precaution against backflow, the needle remained in place for an additional 10 min and was then slowly removed. Mice in both the ICH and BYHWD groups received the autologous blood injection procedure described above. The sham-operated group had the same type of empty needle inserted in the same way at the same site. Mice in the BYHWD group were administered BYHWD (43.29 g/kg) once a day over for 7 days. An equal volume of double-distilled water was used as the control for mice in the ICH and sham groups. Body weight was assessed before and on days 1, 3, and 7 post-modelling. Finally, all animals were sacrificed on day 7, and the whole brain or brain tissue surrounding the hemorrhagic region was harvested and stored for experimental evaluation. Failed models and mice that died were excluded.

### Behavioural test

The survival status of the animals was recorded daily and Kaplan–Meier curves were used to express the survival rate. Neurologic deficits were assessed using the modified neurological severity score (mNSS) and foot fault test. The mNSS is based on motor, reflex, and balance tests, which are rated on a scale from 0 to 18. The higher the neurological function score, the more severe the neurological deficit. The lowest score was taken after three independent replicate experiments for each animal. To evaluate sensorimotor function, the foot fault test was performed 1, 3, and 7 days after modelling. Data are presented as the percentage of left foot faults per the total number of both left and right steps (normal score, 0%; maximal deficit score, 100%). The results of the pre-modelling behavioural tests were used as baseline levels, with higher scores indicating more severe neurological impairments.

### Haematoxylin and eosin (H&E) staining

The paraffin sections of brain tissue were baked in an oven at 60 °C for 1 h. Next, they were soaked in xylene solution for 15 min and then replaced with clean xylene and soaked again for 15 min, shaking continuously throughout to ensure complete wax removal. For hydration, the sections were immersed in a gradient of concentrations containing alcohol, in the order of 100%, 100%, 95%, 85%, and 75%, for 5 min, respectively. The haematoxylin staining solution (Servicebio, China) was used to immerse the sections for approximately 10 min, followed by rinsing under running water for 5 min to remove floating colours. Next, the tissue sample was placed in hydrochloric acid ethanol for 3 s till it turned blue. Afterward, we immersed the sections in an eosin staining solution (Servicebio, China) for approximately 5 min. The sections were then dried naturally and sealed with neutral resin. Histopathological examinations were performed using a light microscope (Axio Image M2, ZEISS, Germany) at 25 °C according to the manufacturer’s instructions.

### Nissl staining

For preparation, 4-μm coronal brain sections were deparaffinised in xylene, rehydrated with ethanol, and stained in Nissl Staining Solution (Servicebio, China) for 5 min at 60 ℃. Each section was deparaffinized in xylene. A light microscope (Axio Image M2, ZEISS, Germany) was used for observation.

### mRNA microarray

Transcriptomic experiments were performed using microarrays to measure mRNA abundance. Briefly, total RNA from the right globus pallidus was extracted and purified using the RNeasy Mini Kit (Qiagen, USA). Tissues were prepared, and chips were hybridised using the Agilent Gene Expression Hybridisation Kit (Agilent Technology, USA). After washing, the arrays were scanned with an Agilent chip reader and analysed using the Agilent feature extraction software (version 10.5.1.1.1.1.1). Comparing the ICH group with the sham group and the BYHWD with the ICH group, differentially expressed transcripts and mRNAs were revealed (Fold Change, FC ≥ 1.3, *P* < 0.05). The mRNA expression in 15 mice (three groups with five replicates) was detected using microarrays.

### Bioinformatics analysis

Gene Ontology (GO; containing biological process, cellular component, and molecular function and Kyoto Encyclopaedia of Genes and Genomes (KEGG)) was conducted to determine the biological annotation of targets using Metascape (http://metascape.org).

### Construction of the network pharmacology

Candidate bioactive ingredients of seven herbs were screened from the Traditional Chinese Medicine Systems Pharmacology (TCMSP) database (http://lsp.nwu.edu.cn/tcmsp.php) and the BATMAN-TCM (http://bionet.ncpsb.org/batman-tcm/) database and searched in the PubMed database. Only chemicals with an oral bioavailability ≥ 30 and a drug-likeness ≥ 0.18 that met the criteria recommended by the TCMSP database were retained. The name of the identified compound was input into the TCMSP database, SEA database (SEA Search Server bkslab.org), and Swiss Target Prediction (http://www.swisstargetprediction.ch/) to obtain its target name. The target name was used to convert to the target gene and confine the species to Homo sapiens in the UniProt (http://www.uniprot.org/) database. Information on ICH-associated target genes was collected from GeneCards (http://www.genecards.org/), the OMIM database (http://www.omim.org/), Therapeutic Target Database (TTD) (http://db.idrblab.net/ttd/), and Drugbank (https://go.drugbank.com/) database. The drug-protein-target and the disease-drug-target networks were constructed using Cytoscape 3.6.1.

### Real-time quantitative polymerase chain reaction (RT-qPCR)

RT-qPCR assays were used to detect the amplification of specific genes. The total RNA from the basal ganglia of the mouse brain was reverse transcribed into cDNA using a first-strand synthesis kit (Vazyme, China) according to the manufacturer’s instructions. A CFX Connect fluorescent quantitative PCR system (Bio-Rad, USA) was used for RT-qPCR amplification. The cycling procedure was as follows: incubation at 95 °C for 3 min, followed by 40 cycles of 95 °C for 10 s and 55 °C for 30 s. Relative expression was calculated as 2^−ΔΔC^. GAPDH was used as the internal reference. Specific primer sequences are listed in Table 2.

**Table 2:** Sequences of primers for RT-qPCR validation

Gene name	Forward and reserve primer (5′—3′)
Tlr4	F:TGTGTCAGTGGTCAGTGTGATTGTG R: CTGTAGTGAAGGCAGAGGTGAAAGC
Abcb1b	F: TATCCCACCCGACCCAACATCC R:GCTGCCCTCACAATCTCCTCATG
Birc5	F: CTACCGCATCGCCACCTTCAAG R: GGCTCTCTGTCTGTCCAGTTTCAAG
Ccl12	F: AGCTACCACCATCAGTCCTCAGG R: TCTCCTTGGGGTCAGCACAGATC
Ctsb	F: GGTTGCGTTCGGTGAGGACATAG R: AGGGAGGGATGGTGTATGGTAAGC
Mcoln1	F:AACACCATTGCCTTCCGACATCTC R:CCATCCAAGAAGTCCAAGTCCTCAC
Tfeb	F: AGCAGGTGGTGAAGCAAGAGTTG R: GGGAGCAGGGAGTCATCTAGGAG
Gapdh	F:TCACCATCTTCCAGGAGCGAGAC R: TGAGCCCTTCCACAATGCCAAAG
Rab7b	F:ACAAAGGTTCCGATGGCTGTATCC R: TGGAGTCTATGAGGTGGTTCTCTGC
Scara5	F:CACCGTGAGTGACCGTGACAAC R:GCATCCTGAACCTTCCACACCTG
Hfe2	F: TTCTCCGCAGAGCAGGACCTAC R: GAAGCAAAGCCACAGAACAAAGAGG
Trf	F: TGAACTGCTCTGCCTTGACAATACC R:CACACGCCTTCCTGCTGATTCC
Pycard	F:GCAACTGCGAGAAGGCTATGGG R:CTCATCTTGTCTTGGCTGGTGGTC

*F* forward primer, *R* reverse primer

### Immunofluorescence

After immersion in xylene and alcohol, antigen repair was performed using sodium citrate buffer (pH = 6.0) for thermal repair, 0.1 M phosphate-buffered saline (PBS, pH 7.4) for three washes, 3% BSA to block non-specific antigen, followed by overnight incubation of the sections with anti-ATG5 (1:5000, Huabio, China) at 4 °C. Subsequently, the sections were incubated with the secondary antibody donkey anti-mouse Cy3 (1:1000, Jackson ImmunoResearch, USA) for 1 h at 25 °C. Following incubation, the cell nuclei were stained with DAPI (1:50, Solarbio, China). Finally, paraffin sections were covered with an anti-fluorescent quencher (Servicebio, China).

### Western blotting

The protein concentrations of the samples were determined using a BCA Protein Assay Kit (Thermo, USA). Then, the samples were mixed with a fourfold loading buffer, boiled for 5 min, and stored at − 80 °C until use. Equal amounts of protein (30 μg) were electrophoresed on 10% polyacrylamide gel with sodium dodecyl sulphate (Applygen, China). After gel electrophoresis, the target proteins were transferred to polyvinylidene difluoride membranes (Millipore Corp., Billerica, USA) using WB transfer buffer (Applygen, China) and fixed with 5% skimmed milk on a shaker for 2 h. The membranes were then incubated with the primary antibody overnight at 4 °C. After washing three times with Tris-BufferedSalineTween20, the membranes were incubated with an HRP-labelled secondary antibody for 1 h at 25 °C. The PVDF membrane was then immersed in an electrochemiluminescence solution (ECL, Vazyme, China), avoiding light. Finally, the intensity of the bands was quantified using ImageJ software (Rawak Software, Germany). The ratio between the grey scale values of the target band and the internal reference band was used as the relative protein expression. The following primary antibodies were used: anti-ATG5 (1:5000, Huabio, China), anti-Beclin 1 (1:5000, Huabio, China), anti-cathepsin B (1:1000, Huabio, China), anti-p62 (1:1000, Huabio, China), and lc3b rabbit pAb (1:1000, Zenbio, China), with GAPDH (ABCAM, UK) as an internal reference. HRP-conjugated goat anti-rabbit IgG (1:10,000, Jackson ImmunoResearch, USA) and HRP-conjugated goat anti-mouse IgG (1:10,000, Jackson ImmunoResearch, USA) were used as secondary antibodies. All primary antibodies were diluted with WB primary antibody diluent (Beyotime, China). The reaction was performed using ECL reagents. The band density was visualised and analysed using Quantity One software (Bio-Rad, USA).

### Transmission electron microscopy (TEM)

Brain tissue samples were fixed overnight in an electron microscope fixation liquid (Servicebio, China). After washing with PBS, samples were post-fixed in 1% osmium tetroxide in PBS for 2 h. Then, the samples were briefly washed with PBS, dehydrated in an ethanol series (30–100%), and embedded in London Resin (LR) white resin (Taab, Aldermaston, Berks, UK) for polymerisation. Ultrathin Sects. (60–80 nm thick) were cut using a diamond knife on a Reichert Ultracut ultramicrotome (Reichert Company, Vienna, Austria). Subsequently, sample morphology was identified using a transmission electron microscope (Hitachi, Japan) operating at 80 kV.

### Statistical analysis

All quantitative statistical results are presented as the mean ± standard error. A two-way ANOVA was used to analyse the results of the behavioural tests; the quantitative results of the Nissl staining and WB were analysed by one-way ANOVA, followed by the Student–Newman–Keuls test and Dunnett’s test (for comparison of multiple experimental treatments to a common control value). Survival analyses were conducted using the log-rank test. All statistical analyses and plots were performed using GraphPad Prism 8.0. A *p*-value < 0.05 indicated statistical significance.

## Results

### Neuroprotective effects of BYHWD on ICH

**Fig. 1:**
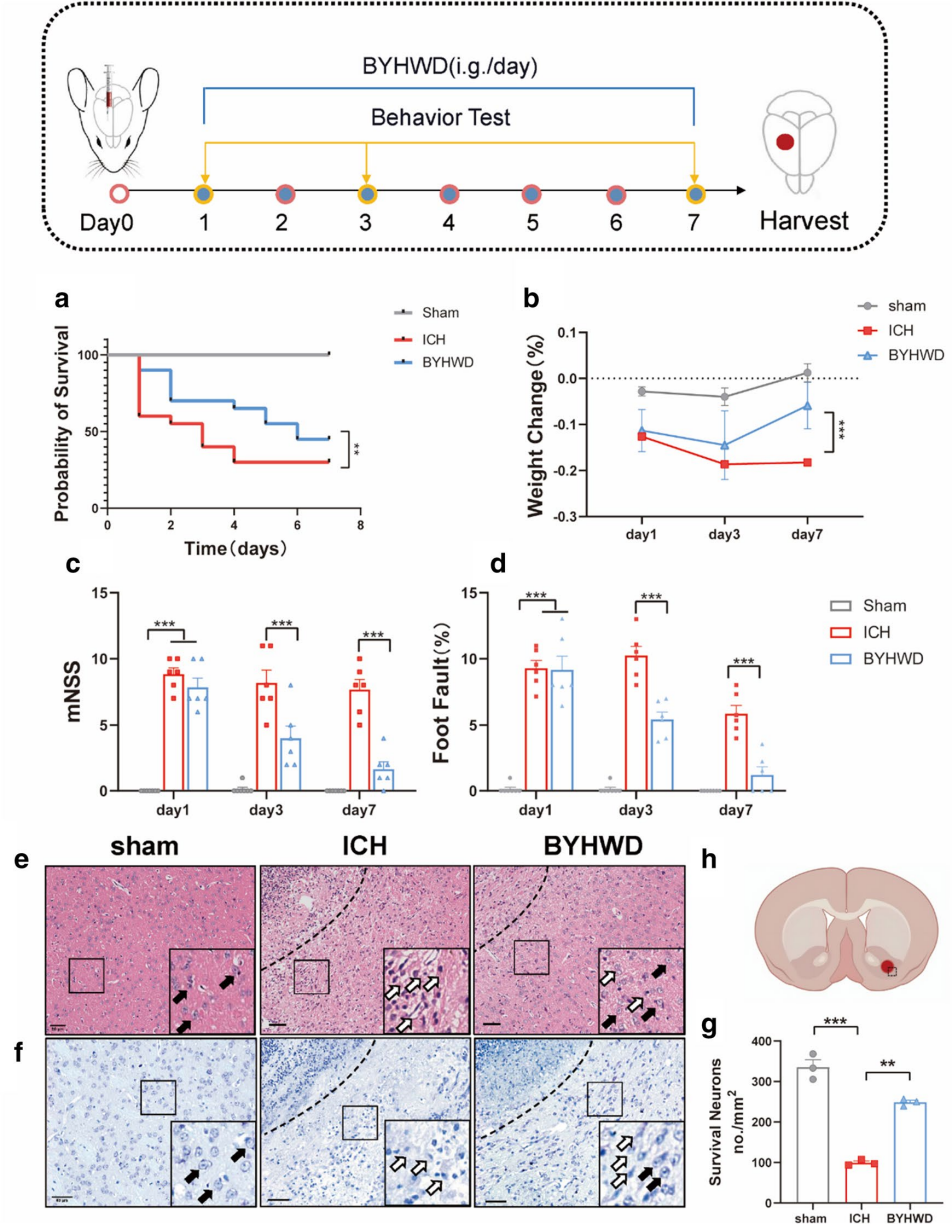
Experimental scheme for ICH models and BYHWD administration. Mice were gavaged (i.g.) with BYHWD (43.29 g/kg) after ICH for 7 days. Kaplan–Meier survival curve assay **a**, body weight changes **b**, modified neurobehavioral scores mNSS **c** and Foot-Fault rate **d** after ICH (n = 6). Representative HE staining **e**, Nissl staining images **f** and quantitative analysis of Nissl staining were performed **g** (n = 3, Scale bar, 50 μm). The dashed line in the figure represents the hematoma area, and the magnified image in the bottom right black box shows the local enlargement of the left side. Solid arrows indicate cells with normal morphology, while hollow arrows represent damaged cells with altered morphology and disorganized arrangement. The sample and pathological observation position of mouse brain in HE staining and Nissl staining **h**. **P < 0.01 ***P < 0.001

All the experimental mice were weighed and analysed survival rates (Fig. 1a) to assess their general conditions (Fig. 1b). After the induction of the ICH model, both the ICH and BYHWD groups showed a decrease in weight growth rate compared to the sham group. However, after drug intervention, the BYHWD group displayed a significantly higher weight growth rate than the ICH group. We utilised the mNSS (Fig. 1c) and the foot fault rate (Fig. 1d) as measures of neurological deficit. The sham group consistently showed low scores at all time points, serving as the control. In contrast, the ICH and BYHWD groups exhibited higher scores on day 1 after ICH (*P* < 0.001), indicating significant neurological insufficiencies. The BYHWD group demonstrated a significant decrease in scores on days 3 and 7, whereas the ICH group maintained higher mNSS scores (*P* < 0.001). The foot fault rate test exhibited a similar trend. The results suggested that BYHWD partially ameliorates neurofunctional default in mice after ICH.

H&E staining was employed to investigate the histopathological changes in the surrounding cellular tissue of the injury site. The results in Fig. 1d revealed that the sham group had orderly cell arrangement and clear morphological features in the cerebral tissue. In contrast, the ICH group exhibited disorganised cell arrangement, swollen cells, and altered intercellular spaces surrounding the haematoma. However, oral administration of BYHWD for 7 consecutive days partially restored the cellular structures near the injury site compared to the ICH group. Brain slices were obtained for Nissl staining for evaluating the impact of BYHWD on the neurons in mice. Following ICH induction, the number of normal Nissl bodies decreased, with unclear and fragmented shapes, suggesting damage to nerve cells (Fig. 1e–f). Conversely, BYHWD significantly alleviated this injury (*P* < 0.05). The findings indicated that BYHWD exhibits a partial protective effect against the pathological destruction observed in ICH mice.

### mRNAs altered in the ICH mouse brain treated by BYHWD

**Fig. 2:**
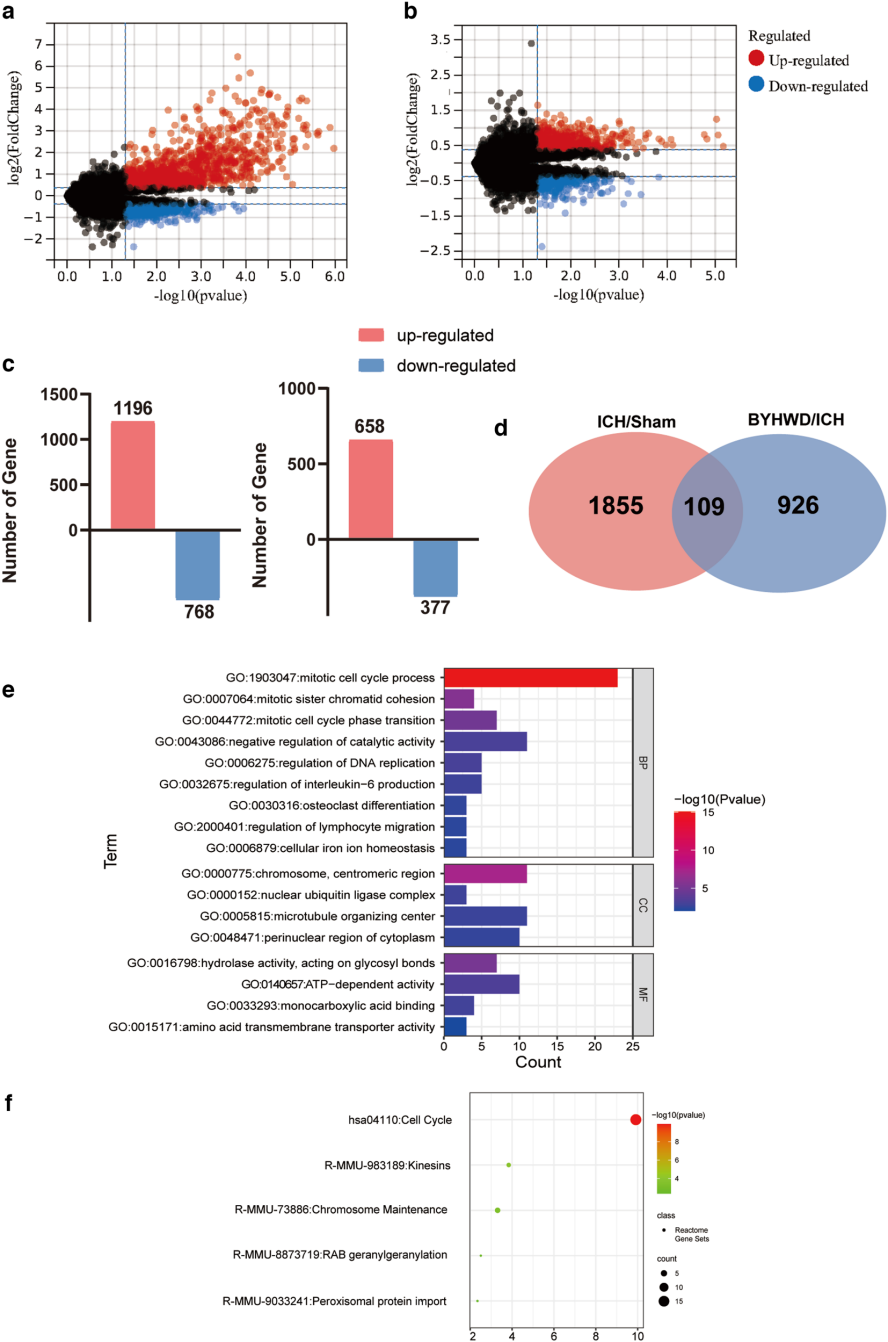
Volcano plots showed differentially expressed mRNAs in ICH/sham groups **a** and BYHWD/ICH groups **b** (with the standard of fold change ≥ 1.3 and* P* < 0.05). Total number of identified differential expression mRNAs **c**. Venn diagram of mRNAs and their overlap **d**. Gene Ontology **e** and KEGG pathway **f** enrichment of mRNAs

Next, Illumina RNA sequencing was used to decipher the function of BYHWD in ICH. A total of 2 999 differentially expressed mRNAs were identified. Specifically, 1 964 mRNAs were identified in the ICH/sham group (1196 were upregulated and 768 were downregulated), and 1 035 mRNAs were identified in the BYHWD/ICH group (658 were upregulated and 377 were downregulated) (Fig. 2a–c). Venn diagrams were used to analyse the overlap between the ICH/sham and BYHWD/ICH groups (Fig. 2d). Consequently, 109 mRNAs were regulated by ICH and reversed after drug intervention (*P* < 0.05, FC ≥ 1.3). To further explore their function, GO and KEGG enrichment analyses were conducted (Figs. 2e–f). GO annotation results were categorised into three groups: cellular components, molecular functions, and biological process annotations. Cellular components included chromosomes, centromeric regions, nuclear ubiquitin ligase complexes, microtubule-organising centres, and perinuclear regions of the cytoplasm; molecular function components included hydrolase activity, acting on glycosyl bonds, ATP-dependent activity, monocarboxylic acid binding, and amino acid transmembrane transporter activity; and biological processes included the mitotic cell cycle process, mitotic sister chromatid cohesion, mitotic cell cycle phase transition, negative regulation of catalytic activity, regulation of DNA replication, regulation of interleukin-6 production, osteoclast differentiation, regulation of lymphocyte migration, and cellular iron ion homeostasis. Based on differential mRNAs, five significant Reactome Gene Sets were identified (*P* < 0.05), including cell cycle, kinesins, RAB geranylgeranylation, peroxisomal protein import, and chromosome maintenance.

### Combined network pharmacology and transcriptomic assay for differential target analysis

**Fig. 3:**
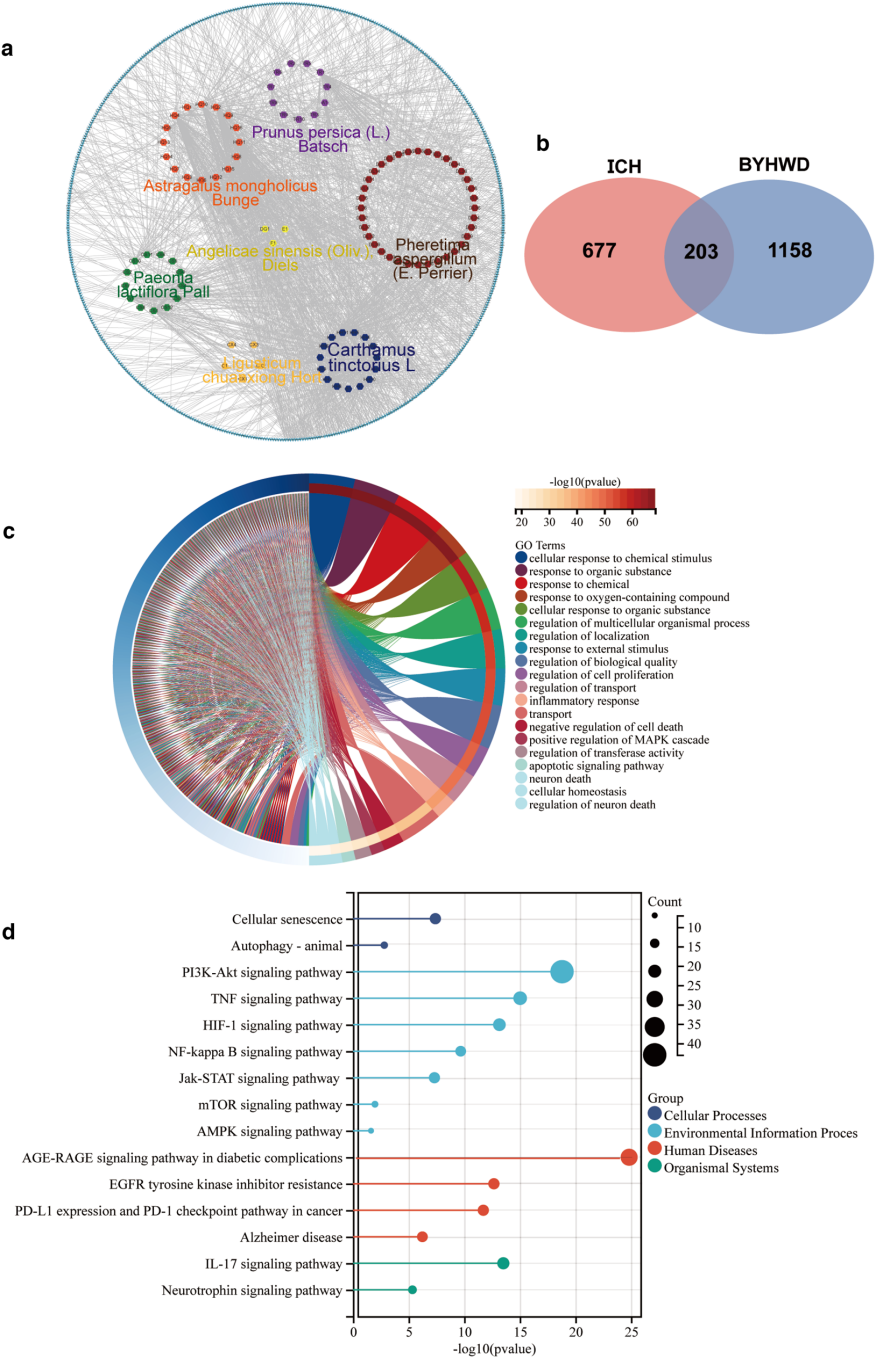
Herbs-Compounds-Targets network of BYHWD **a** displayed the overlap of mRNAs by RNA sequencing and candidate targets identified by network pharmacology **b**. Gene Ontology **c** and KEGG analysis **d** of network pharmacology candidate targets

Network pharmacology was conducted to provide a more accurate analysis. Consequently, 1 361 herb targets were identified. The potential TTD, GeneCards, OMIM database, and Drugbank database disease phenotypes were anchored with ICH, and 880 disease targets were identified. According to the Herbs-Compounds-Targets network constructed using Cytoscape, 653 nodes and 1 629 relation pairs were included (Fig. 3a). The Venn diagram intersection showed that 203 genes overlapped between the drug and disease groups (Fig. 3b). GO and KEGG pathway enrichment analyses were performed to clarify their functions (Fig. 3c–d). GO biological process analysis revealed that the target genes were involved in diverse biological processes, cellular components, and biological processes. Specifically, they were mainly associated with cellular response to chemical stimuli, regulation of multicellular organismal processes, inflammatory responses, apoptotic signal pathways, cellular homeostasis, and regulation of neuronal death. In the KEGG pathway analysis, the targeted genes primarily interacted with the PI3K-Akt, TNF, HIF-1, NF-kappa B, Jak-STAT, mTOR, AMPK, and IL-17 signal pathways. Cellular senescence, autophagy, and Alzheimer’s disease pathways were observed to better understand the potential effects of BYHWD on ICH.

**Fig. 4:**
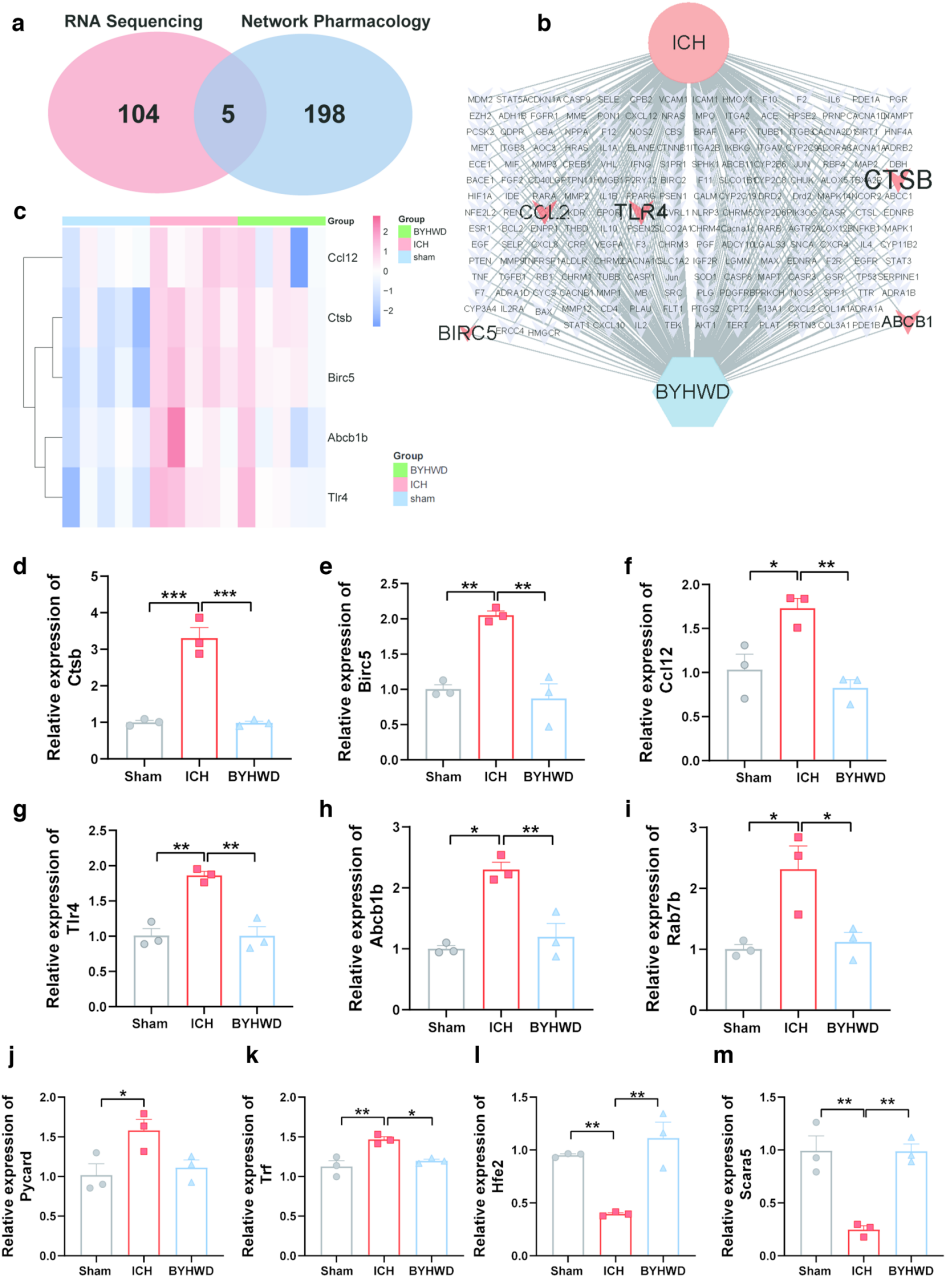
Differential expressed genes of Combined network pharmacology and Illumina sequencing display in Venn Diagram **a**. Disease-Drug-Targets network analysis of BYHWD and ICH **b**. Heat map illustrating target mRNAs **c**. RT-qPCR is performed to validate the results of Illumina sequencing **d**–**m**. n = 3, *P < 0.05 **P < 0.01 ***P < 0.001

Next, omics and network pharmacology analyses were integrated to identify key targets of BYHWD in ICH (Fig. 4a). The Venn diagram illustrates the five overlapping targets: cathepsin B (Ctsb), ATP binding cassette subfamily B member 1 (Abcb1b), toll-like receptor 4 (Tlr4), chemokine (C–C motif) ligand 12 (Ccl12), and baculoviral IAP repeat-containing 5 (Birc5). Disease-drug-target network analysis of BYHWD and ICH was performed to visualise the link between disease and drugs (Fig. 4b). The clustering heatmap (Fig. 4c) was used to observe trends among the three groups. To verify the results derived from bioinformatics analysis, haemojuvelin (Hfe2), scavenger receptor class A member 5 (Scara5), Ras-related protein Rab-7b, transferrin, PYD and CARD Domain Containing (Pycard), Ccl12, Abcb1b, Tlr4, Ctsb, and Birc5 were randomly selected for RT-qPCR (Fig. 4d–l). In particular, Abcb1b, Birc5, Ccl12, Trf, Tlr4, Rab7b, Pycard, and Ctsb expressions were upregulated after ICH; however, a significant decrease was observed after BYHWD treatment. The expression of Hfe2 and Scara5 demonstrated opposite trends in that they were downregulated after ICH but upregulated after BYHWD treatment. The results of the experiment were consistent with the microarray results. Among the five core targets, Ctsb showed the most significant change in mRNA expression (*P* < 0.001).

### BYHWD enhances autophagy by regulating Ctsb in ICH

**Fig. 5:**
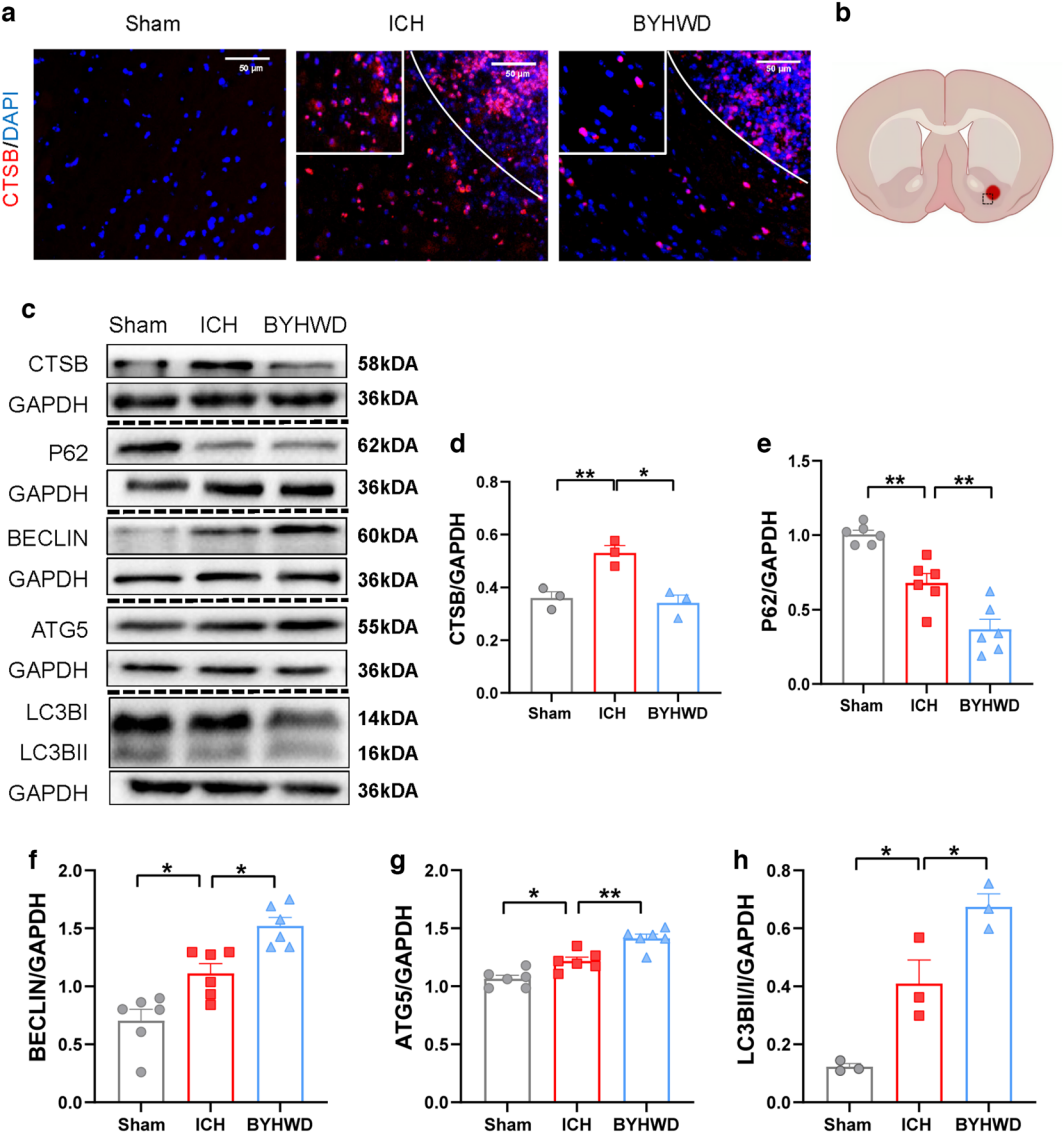
Immunofluorescence was utilized to detect Ctsb-positive cells in different groups, the white solid line in the figure represents the hematoma area, and the magnified image in the top left white box shows a local enlargement **a**. The sample and pathological observation position of mouse brain in immunofluorescence **b**. Western blotting (WB) was conducted to assess the levels of Ctsb and autophagy-related molecules **c**. The quantitative analysis in WB of CTSB **d**, P62 **e**, BECLIN **f**, ATG5 **g** and LC3B **h**, n = 3–6, *P < 0.05 **P < 0.01

**Fig. 6:**
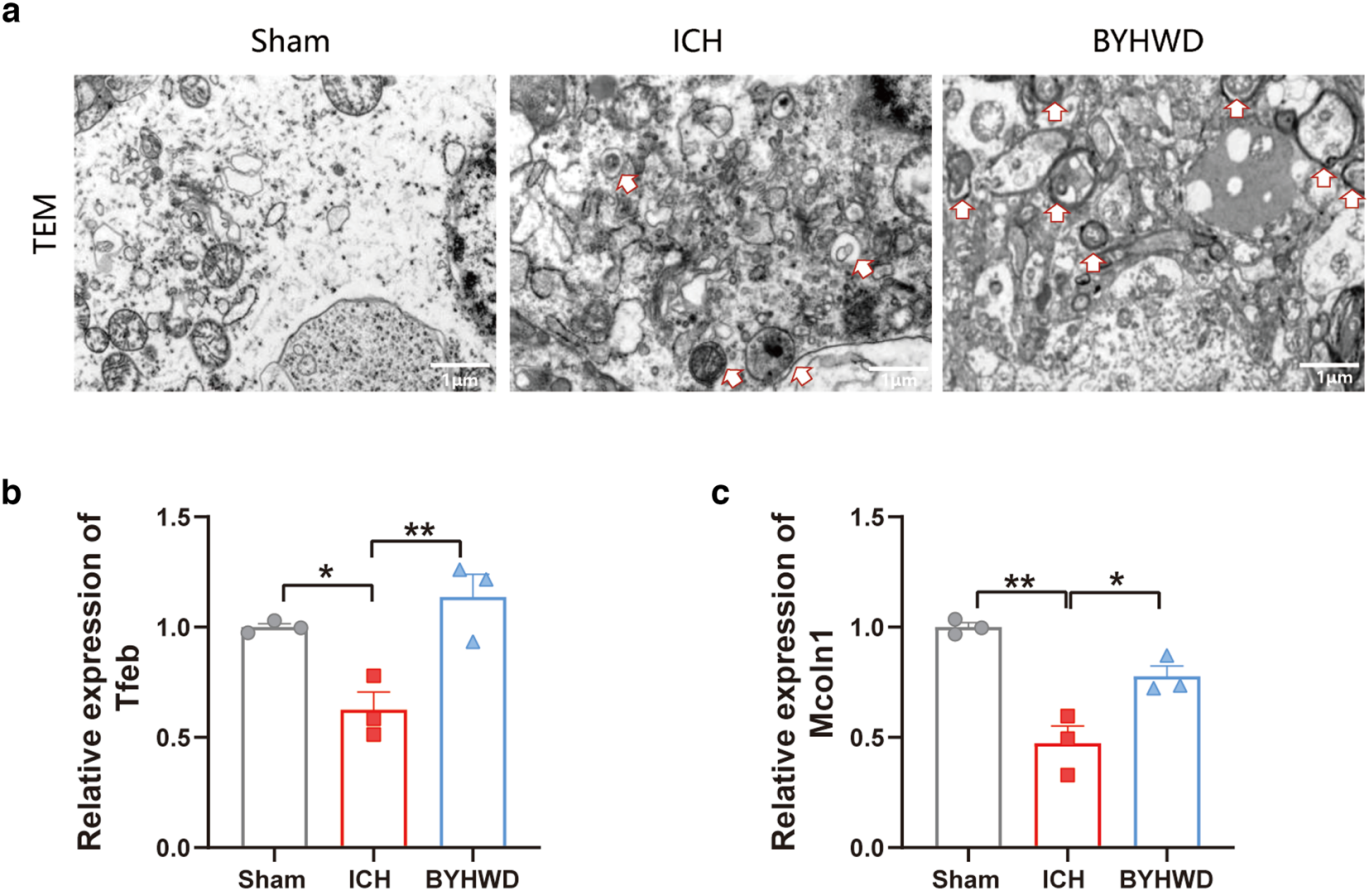
A transmission electron microscopy image was obtained to visualize autophagosomes. The white arrows refer to autophagosomes. **a**. RT-qPCR analysis was used to examine the mRNA expression levels of Tfeb **b**, and Mcoln1 **c**. n = 3, *P < 0.05 **P < 0.01 ***P < 0.001

As shown in Fig. 4d, the significant change in mRNA expression of Ctsb was also visualised using immunofluorescence and western blot (Fig. 5a–c). The increase in Ctsb expression following ICH was reduced after BYHWD treatment. Due to its localisation on lysosomes, Ctsb may potentially influence the autophagic process [34]. Western blotting was used to assess changes in autophagy-related proteins to further investigate the potential mechanism of BYHWD regulation of Ctsb (Fig. 5b). After ICH on day 7, the expression of the autophagy substrate P62 (Fig. 5d) decreased in the ICH group than in the sham group, whereas the expression of autophagy-related proteins such as Beclin1, Atg5, and Lc3b increased (Fig. 5e–g). The trends were reinforced in the BYHWD group. Autophagosomes were observed by TEM, the gold standard for studying autophagy. The experimental results suggested that autophagic activity was active and autophagy levels were enhanced after BYHWD treatment (Fig. 6a). Ctsb cleaves the adhesive protein 1 (mucolipin 1, Mcoln1) in lysosomal calcium channels, inhibiting the synthesis of transcription factor EB (Tfeb) and reducing the expression of lysosomal and autophagy-related proteins, thus impacting cellular autophagy [35]. The mRNA expression of Tfeb (Fig. 6b) and Mcoln1 decreased in the ICH group but increased in the BYHWD group (Fig. 6c), which was negatively correlated with the mRNA expression of Ctsb. These experiments suggest that BYHWD exerts a neuroprotective effect by inhibiting Ctsb and enhancing the expression of Tfeb and Mcoln1 to increase autophagy.

## Discussion

In this study, we identified five critical differentially expressed mRNAs following BYHWD intervention after ICH using a transcriptomic-combined network pharmacology strategy. Among these mRNAs, the most prominently altered mRNA was Ctsb, which acts therapeutically by enhancing autophagy. We have attempted to elucidate the possible mechanisms of the pharmacotherapeutic efficacy of BYHWD for ICH treatment and to achieve precise therapy at the transcriptional level.

Previous studies have shown that the administration of BYHWD can attenuate neuronal death and improve neurological deficits after ICH [27, 32]. In this study, BYHWD significantly enhanced the recovery of neurological function defects, as determined by several classical behavioural tests. Furthermore, BYHWD effectively reversed the neurofunctional and pathological impairments induced by ICH. These findings underscore the crucial role of BYHWD in exerting a neuroprotective response against ICH.

The mRNA alterations caused by BYHWD treatment are a complex process. Five differentially expressed mRNAs, namely Abcb1b, Ccl12, Birc5, Tlr4, and Ctsb, overlapped (Table 3). Our research confirmed an association between Abcb1b and BYHWD on ICH. Abcb1b is an ATP-dependent drug efflux pump that maintains intracellular homeostasis and belongs to subfamily B of the ATP-binding cassette transporter superfamily. In the nervous system, Abcb1b is present in the blood–brain barrier (BBB), where it restricts drug penetration into the brain [36, 37]. Recent clinical cross-sectional findings have suggested an association between two SNPs in Abcb1b and post-thrombolytic hemorrhagein ischaemic stroke [38]. However, the specific mechanism of action remains unclear. Our results indicated that the crucial role Abcb1b plays may be attributed to the modulation of drug penetration across the BBB by affecting proton pump activity. Inhibitor of apoptosis (IAP) repeat containing 5 (Birc5), also known as survivin, is a member of the IAP family and is highly expressed in most tumor cells [36]. Birc5 negatively regulates apoptosis by inhibiting the expression of caspase 3 [37]. The therapeutic effect of BYHWD on ICH may be attributed to its ability to induce apoptosis through Birc5 downregulation. Ccl12 belongs to the C–C (or beta) chemokine family, which modulates immune regulation and inflammation [38]. During ICH, Ccl12 induces inflammation and exacerbates brain damage by recruiting macrophages and T cells [39]. In this study, BYHWD intervention reversed the upregulation of Ccl12 after modelling.

**Table 3:** Differentially expressed genes regulated by BYHWD

Transcript ID	Mouse gene	Fold Change (ICH/Sham)	Fold Change (BYHWD/ICH)	Description	Human gene
ENSMUST00000048096	Tlr4	0.27	1.87	Toll-like receptor 4	TLR4
ENSMUST00000000194	Ccl12	0.72	1.62	Chemokine (C–C motif) ligand 12	CCL2
ENSMUST00000009058	Abcb1b	0.72	1.33	ATP-binding cassette, sub-family B (MDR/TAP), member 1B	ABCB1
ENSMUST00000081387	Birc5	0.19	1.74	Baculoviral IAP repeat-containing 5	BIRC5
ENSMUST00000006235	Ctsb	0.33	1.36	Cathepsin B	CTSB

The neuroprotective effect of BYHWD may be achieved by reducing the expression of Ccl12, resulting in the inhibition of inflammation. Tlr4 is an innate immune receptor belonging to the toll-like receptor family [40, 41]. Tlr4 is a marker of inflammation and apoptosis in ICH and is increased following secondary brain injury. In turn, high Tlr4 expression exacerbates neurological defects, consistent with our results [42]. We speculate that BYHWD downregulates Tlr4 expression to alleviate neuroinflammation and improve the prognosis of patients with ICH. In conclusion, although we have confirmed the association between key genes such as Abcb1b, Birc5, Ccl12, Tlr4, and Ctsb and BYHWD treatment for ICH in our study. Further experimental validation is still required to elucidate their functional significance and mechanisms of action in ICH pathophysiology. The specific roles and interrelationships of these genes are crucial for understanding the pathogenesis of ICH and the therapeutic effects of BYHWD.

The most significant alteration in gene expression following BYHWD intervention in ICH was observed in Ctsb during the study. Cathepsin B, a member of the cysteine cathepsin family, is primarily located in lysosomes and endosomes. It plays a role in autophagy, antigen presentation, cellular stress signalling, and lysosome-dependent cell death [43]. Previous studies have reported that the deletion of the Ctsb gene has a significant positive impact on behavioural deficits in conditions such as ischaemic neuropathic damage, inflammatory pain, opioid tolerance, epilepsy, and Alzheimer's disease [44]. Additionally, research has indicated the involvement of Ctsb in neuronal cell death following ICH. Selective Ctsb inhibition improves neurological function in rats, potentially by promoting cell survival at the ICH border and restoring neuroplasticity and angiogenesis [45]. Ctsb directly activates several matrix metalloproteinases (MMPs), and its absence inhibits MMP-9 expression [35]. MMP-9 is associated with ICH enlargement and the development of perihaematomal oedema [46]. Interestingly, a previous study revealed that Ctsb plays a role in correct chromosome segregation, which is closely related to lysosomal function [47]. This finding aligns with the biological enrichment results of transcriptomics analysis, specifically highlighting the involvement of Ctsb in chromosomal maintenance. Collectively, these findings support the notion that Ctsb is involved in neural restoration following ICH through multiple pathways. This further underscores the potential of Ctsb as a critical target for BYHWD therapy. The localisation of Ctsb within lysosomes suggests its potential impact on the autophagy process [34]. Autophagy plays a vital role in degrading intracellular materials and maintaining cellular homeostasis by utilising lysosomal enzymes [48]. The production and transportation of autophagosomes primarily occur in axons, and proper axonal transport is crucial for clearing damaged mitochondria [19]. Ctsb is closely associated with autophagy and negatively regulates neurite outgrowth by modulating lysosomal trafficking in neurons [49]. In our study, autophagy levels were elevated following ICH injury. However, concomitant increases in both Ctsb mRNA and protein expressions suggest that Ctsb may not be the sole factor influencing autophagy levels, as the autophagy process is regulated by multiple factors [50]. Conversely, after intervention with BYHWD, a significant enhancement in autophagy levels was observed, accompanied by a decrease in Ctsb levels. This indicates that BYHWD can partially inhibit Ctsb expression and enhance autophagy.

Under normal homeostatic conditions, Ctsb cleaves Mcoln1 within the lysosome, inhibiting calcium efflux from the lysosomal lumen to the cytoplasm. This process restricts the expression of Tfeb and subsequently reduces the expression of lysosomal and autophagy-related proteins [34]. Tfeb is a key regulator of autophagy and lysosomal biogenesis [51], and its activation alleviates neurodegeneration by promoting lysosomal function and inducing autophagy [52, 53]. Mcoln1 is a ubiquitously expressed lysosomal calcium channel, which is activated during autophagy and is involved in lysosomal calcium transport [54]. Furthermore, the Mcoln1/calcineurin-dependent mechanism is implicated in autophagy initiation [52].

In our study, the mRNA expression levels of Mcoln1 and Tfeb decreased after BYHWD intervention. Thus, we hypothesised that BYHWD moderates the expression of Ctsb, Mcoln1, and Tfeb to regulate autophagy after ICH. Autophagy plays a bidirectional role in ICH. Some studies have demonstrated that autophagy is involved in neuronal death following brain injury [55]; others have revealed that enhancing autophagy 7 days after ICH improves disease prognosis [21]. However, the integrity of autophagic flux and the autophagy level are critical factors that influence the effectiveness of autophagy [56].

Basic experimental research has suggested that in the early stages after injury, many autophagic vesicles around the haematoma show impaired autophagic flux and production of cellular waste. At this point, high levels of autophagy and endoplasmic reticulum stress are in a positive feedback loop, exacerbating nerve damage. However, 7 days after ICH, the autophagic flux returns to normal, allowing autophagy to efficiently clear the metabolic waste and play a neuroprotective role [57]. These studies collectively demonstrate that cellular autophagy activation has a beneficial effect 7 days post-ICH. In summary, the therapeutic mechanism of BYHWD at 7 days after ICH likely involves enhancing cellular autophagy through the downregulation of Ctsb expression.

## Conclusions

In summary, based on a combination of bioinformatics analyses and validation experiments, we have elucidated the key target proteins and specific mechanisms of BYHWD treatment for ICH from multiple perspectives, which are associated with apoptosis, signal pathways, and PI3K-Akt/mTOR biological processes. In addition, we confirmed that BYHWD improves the prognosis of ICH by inhibiting Ctsb and enhancing cell autophagy.

## Data Availability

The data used to support the findings of this study are available from the corresponding author upon request.

## Abbreviations

ICH: Intracerebral hemorrhage
BYHWD: Buyang Huanwu Decoction
mNSS: Modified neurological severity score
H&E: Haematoxylin and eosin
TCMSP: Traditional Chinese Medicine Systems Pharmacology
RT-qPCR: Real-time quantitative polymerase chain reaction
TEM: Transmission electron microscopy
TDD: Therapeutic target database
Ctsb: Cathepsin B
Abcb1b: ATP binding cassette subfamily B member 1
Tlr4: Toll-like receptor 4
Ccl12: Chemokine (C–C motif) ligand 12
Birc5: Baculoviral IAP repeat-containing 5
Tfeb: Transcription factor EB
BBB: Blood brain barrier
IAP: Inhibitor of apoptosis
Birc5: Inhibitor of apoptosis (IAP) repeat containing 5
MMPs: Matrix metalloproteinases
Mcoln1: Mucolipin 1
Hfe2: Haemojuvelin,
Scara5: Scavenger receptor class A member 5
